# Could Artificial Intelligence/Machine Learning and Inclusion of Diet-Gut Microbiome Interactions Improve Disease Risk Prediction? Case Study: Coronary Artery Disease

**DOI:** 10.3389/fmicb.2022.627892

**Published:** 2022-04-11

**Authors:** Baiba Vilne, Juris Ķibilds, Inese Siksna, Ilva Lazda, Olga Valciņa, Angelika Krūmiņa

**Affiliations:** ^1^Bioinformatics Lab, Riga Stradins University, Riga, Latvia; ^2^COST Action CA18131 - Statistical and Machine Learning Techniques in Human Microbiome Studies, Brussels, Belgium; ^3^Institute of Food Safety, Animal Health and Environment BIOR, Riga, Latvia; ^4^Department of Infectology and Dermatology, Riga Stradins University, Riga, Latvia

**Keywords:** machine learning, diet, gut microbiome, personalized nutrition, coronary artery disease, artificial intelligence, risk prediction

## Abstract

Coronary artery disease (CAD) is the most common cardiovascular disease (CVD) and the main leading cause of morbidity and mortality worldwide, posing a huge socio-economic burden to the society and health systems. Therefore, timely and precise identification of people at high risk of CAD is urgently required. Most current CAD risk prediction approaches are based on a small number of traditional risk factors (age, sex, diabetes, LDL and HDL cholesterol, smoking, systolic blood pressure) and are incompletely predictive across all patient groups, as CAD is a multi-factorial disease with complex etiology, considered to be driven by both genetic, as well as numerous environmental/lifestyle factors. Diet is one of the modifiable factors for improving lifestyle and disease prevention. However, the current rise in obesity, type 2 diabetes (T2D) and CVD/CAD indicates that the “one-size-fits-all” approach may not be efficient, due to significant variation in inter-individual responses. Recently, the gut microbiome has emerged as a potential and previously under-explored contributor to these variations. Hence, efficient integration of dietary and gut microbiome information alongside with genetic variations and clinical data holds a great promise to improve CAD risk prediction. Nevertheless, the highly complex nature of meals combined with the huge inter-individual variability of the gut microbiome poses several Big Data analytics challenges in modeling diet-gut microbiota interactions and integrating these within CAD risk prediction approaches for the development of personalized decision support systems (DSS). In this regard, the recent re-emergence of Artificial Intelligence (AI) / Machine Learning (ML) is opening intriguing perspectives, as these approaches are able to capture large and complex matrices of data, incorporating their interactions and identifying both linear and non-linear relationships. In this Mini-Review, we consider (1) the most used AI/ML approaches and their different use cases for CAD risk prediction (2) modeling of the content, choice and impact of dietary factors on CAD risk; (3) classification of individuals by their gut microbiome composition into CAD cases vs. controls and (4) modeling of the diet-gut microbiome interactions and their impact on CAD risk. Finally, we provide an outlook for putting it all together for improved CAD risk predictions.

## 1. Introduction

Coronary artery disease (CAD) is the most common cardiovascular disease (CVD) and the main leading cause of morbidity and mortality worldwide, posing a huge socio-economic burden to the society and health systems (Lopez et al., 2006). Currently, our health care system is facing a paradigm shift from a “one size fits all” approach to a more optimized model to identify prevention strategies and treatments tailored to each individual, the so called personalized medicine. Moreover, the vision of prevention has also transformed toward a concept of “positive health” and primordial prevention—the prevention of disease risk factors before they actually occur, i.e., through targeted modifications of person's environment/lifestyle (Movsisyan et al., 2020). Therefore, timely and precise identification of people at high risk of CAD is of utmost importance for the personalized cardiology (Alaa et al., 2019), as such persons may need more aggressive health promotion strategies, especially the modifiable CAD risk factors could be effectively reduced or even eliminated in this way (Movsisyan et al., 2020).

Over the past two decades, numerous approaches for CAD risk prediction have been developed and several have also entered the clinical routine such as the Framingham Risk Score (FRS) (Wilson et al., 1998) or the Systematic Coronary Risk Evaluation (SCORE) metrics (Conroy et al., 2003), extensively reviewed elsewhere (Damen et al., 2016; Westerlund et al., 2021). However, these approaches are mostly based on a limited number of predictors—the traditional CAD risk factors (age, sex, diabetes, systolic blood pressure, LDL/HDL cholesterol, smoking). Hence, incompletely predictive of disease onset, progression and clinical outcome across all patient groups (Alaa et al., 2019), overestimating the 10-year CAD/CVD risk, especially for high-risk individuals and European populations (Damen et al., 2016). These models typically do not take into account the fact that the treatment options have improved and that, by modifying the person's environment/lifestyle, the disease risk can be reduced over time (Westerlund et al., 2021).

CAD is a multi-factorial disease with complex etiology, considered to be driven by both environment/lifestyle and genetic factors (Davey Smith et al., 2005; Erdmann et al., 2018; Vilne and Schunkert, 2018). Over the last 14 years, several large-scale genome-wide association studies have aimed to identify the genetic factors associated with CAD risk (Samani et al., 2007; Erdmann et al., 2009; Tregouet et al., 2009; Schunkert et al., 2011; Deloukas et al., 2012; Nikpay et al., 2015; Howson et al., 2017; Nelson et al., 2017; Webb et al., 2017; van der Harst and Verweij, 2018) and their functional consequences (Brænne et al., 2015; Kessler et al., 2015, 2016, 2017; Zhao et al., 2016; Aherrahrou et al., 2017; Vilne et al., 2017; Lempiäinen et al., 2018; Schunkert et al., 2018; Neiburga et al., 2021). It is currently a matter of intense debate, whether it might be time to implement genetic variations in the clinical routine CAD risk predictions (Inouye et al., 2018; Khera et al., 2018; Cecile et al., 2019; Gola et al., 2020; Lieb and Vasan, 2020).

At the same time, the contribution of environmental/lifestyle factors, in particular, dietary factors have remained less investigated (Khera et al., 2017; Dimovski et al., 2019). Diet is one of the modifiable factors for disease prevention and dietary recommendations have been formulated for decades to guide us toward changing our eating habits in favor of healthy choices. For example, the consumption of foods abundant in cholesterol and fats, such as (processed) red meats, have been associated with increased CAD risk and mortality (Bernstein et al., 2010; Micha et al., 2010). First evidence suggests that even for individuals at high genetic CAD risk and with pre-existing non-modifiable risk factors (age, sex, positive family history) adherence to a healthy lifestyle could be associated with an almost 50% lower relative risk of CAD (Khera et al., 2017; Dimovski et al., 2019), indicating that the inclusion of dietary factors can substantially improve CAD risk prediction, as compared to standard Cox models without these additional variables (Rigdon and Basu, 2019; Ho et al., 2020). With the advent of biosensors and wearable health technology connected to mobile apps, large-scale longitudinal food diaries and images of meals consumed are increasingly becoming available and are even being integrated within electronic health records (Verma et al., 2018; Dinh-Le et al., 2019; Moraes Lopes et al., 2020), whereas further advances in and rapidly decreasing costs of next generation sequencing generate increasing data volumes describing the human gut microbiome qualitative and quantitative composition and function (Eetemadi et al., 2020), thus providing valuable sources of data for integration in the context of personalized diet recommendation systems (Eetemadi et al., 2020), which could be further integrated into clinical decision support systems for improved CAD risk predictions. However, the current rise in obesity, type 2 diabetes (T2D) and CVD/CAD (Pallazola et al., 2019), indicates that the “one-size-fits-all” approach may not be efficient, due to significant variation in inter-individual responses to diet (Hughes et al., 2019), and that interactions between diet and other factors need to be considered (Qi, 2012).

Recently, the human gut microbiome has emerged as a potential and previously under-explored contributor to these variations (Bashiardes et al., 2018), as the composition and function of this complex community of trillions of microorganisms (including bacteria, archaea, viruses, and microbial eukaryotes) (Garud and Pollard, 2020) is modulated by dietary components, e.g., the well-known beneficial impact of the so called Mediterranean diet (De Filippis et al., 2016). This impact is partly mediated through the metabolization and transformation of different nutrients by the gut microbiome, generating secondary metabolites, with changed retention time, bioactivity and different impact on health outcomes: being either protective, such as the short-chain fatty acids (SCFA) or promoting the disease development such as hydrogen sulfite or bile acids (Ni et al., 2015; Hughes et al., 2019; Eetemadi et al., 2020). Changes in the qualitative and quantitative composition of the gut microbiome have been increasingly linked to a number of diseases, including obesity (Turnbaugh et al., 2009; Maruvada et al., 2017; Miyamoto et al., 2019) and CVD/CAD (Koeth et al., 2013; Miele et al., 2015; Tang et al., 2017; Ascher and Reinhardt, 2018). Hence, efficient integration of dietary factors with the gut microbiome holds a great promise to revolutionize the way diseases are treated, through dietary recommendations and lifestyle changes or even the optimization of our gut microbiome, personalized to each individual and the desired health outcomes (Bashiardes et al., 2018; Eetemadi et al., 2020).

Taken together, the multifactorial and complex etiology of CAD (driven by both genetic and environmental/lifestyle factors), combined with the highly complex nature of meals (containing multiple ingredients and spices) and with the additional complexity and huge inter-individual variability of the gut microbiome (Marcos-Zambrano et al., 2021; Moreno-Indias et al., 2021), resulting in completely different responses to identical meals (Zeevi et al., 2015), urgently calling for more advanced problem-solving approaches. Moreover, with the development of high-throughput omic measurement platforms and digitalization of health records, the field is rapidly entering the Big Data era, as the volumes of these data are increasing exponentially (Stephens et al., 2015) and need to be transformed into valuable knowledge. In this regard, the recent re-emergence of advanced computational data-driven technologies such as Artificial Intelligence (AI)/Machine Learning (ML) approaches are opening intriguing perspectivesfor the integration of omics data (genetic variations, gut microbiome) with additional clinical (Reel et al., 2021) and environmental/lifestyle and the development personalized CAD diagnostics tools (Alizadehsani et al., 2019). AI/ML represent automated approaches that are adaptive and able to capture large and heterogeneous matrices of data extracting meaningful patterns and identifying both linear and non-linear relationships between these high-dimensional input variables and the outcomes (Alaa et al., 2019; Rigdon and Basu, 2019; Bodnar et al., 2020; Moraes Lopes et al., 2020). Especially, Deep Learning (DL) approaches, hold a great promise for future progress due to its capabilities to learn from input raw data, instead of using hand-crafted features that require domain expertise (Ching et al., 2018; Solares et al., 2020).

**Figure 1:**
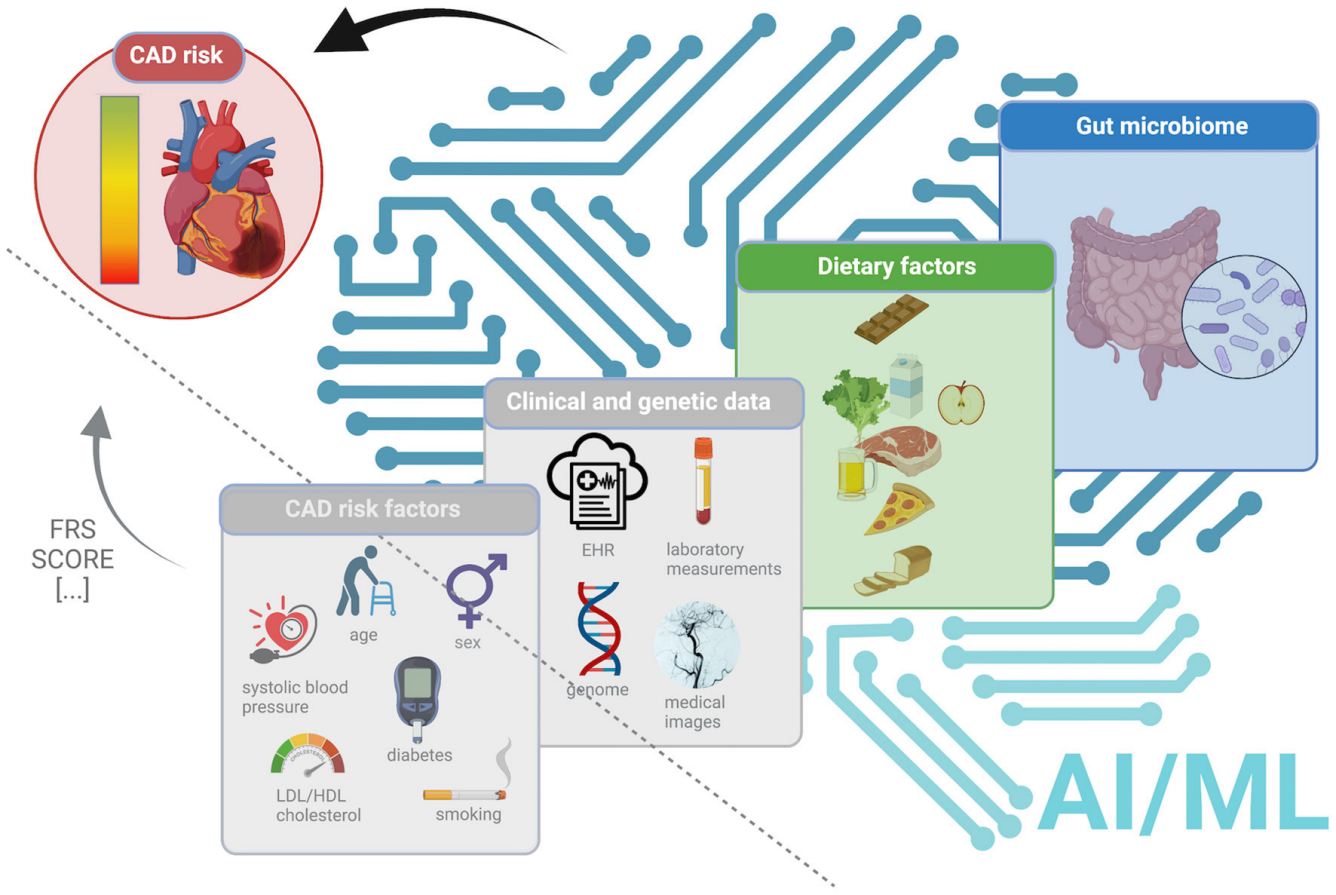
An overview of the current status and future directions to improve CAD risk prediction. The left panel (triangle separated by a dashed line) demonstrates the current status (in gray, as not explicitly considered in this Mini-Review), demonstrates the current metrics such as FRS (Wilson et al., 1998) or SCORE (Conroy et al., 2003) using the traditional CAD risk factors (age, sex, diabetes, systolic blood pressure, LDL/HDL cholesterol, smoking). The right panel (separated by a dashed line) highlights the possible future directions to improve CAD risk prediction using AI/ML approaches and, alongside with clinical data and genetic variations (in gray, as not explicitly considered in this Mini-Review) dietary factors (in green) and/or gut microbiome (in blue).

In this Mini Review, we explore, whether the inclusion of dietary factors and/or gut microbiome data in combination with the power of AI/ML could potentially improve CAD risk prediction. In particular, we consider: (1) the most used AI/ML approaches for CAD risk prediction; (2) the use cases of AI/ML approaches to model the content, choice and impact of dietary factors and how this could be used to predict CAD risk; (3) the use cases of AI/ML approaches to classify individuals by their gut microbiome composition into CAD cases vs. controls and to (4) model the diet-gut microbiome interactions and their impact on CAD risk (as illustrated in Figure 1 and summarized in Table 1). (5) Finally, we provide an outlook for putting it all together into a smart clinical decision support system (DSS), considering the traditional risk factors in combination with individual's genetic variations, as well as dietary factors and gut microbiome and discuss the potential of AI/ML based methods vs. conventional approaches for risk predictions.

**Table 1:** A list of the case studies related to improved CAD risk prediction considered in this Mini-Review.

**Category**	**Study purpose**	**AI/ML approaches(s) used**	**References**
**Dietary factors**	To create an automated mobile vision food diary (Im2Calories), which can recognize the nutritional contents and calories of an individual's meal from its image.	Deep Learning (DL)/Convolutional Neural Network (CNN), adjusted for a mobile phone and images taken “in the wild”	Myers et al., 2015
	Use public food diaries of MyFitnessPal app users to study the food components of a successful (“below” the user defined “daily calories goal”) or un-successful (“above”) diet.	Support Vector Machine (SVM)	Weber and Achananuparp, 2016
	Use the data from the ThinkSlim app, to assess and predict individual's eating behavior in relation to their individual states (location, activity, emotions).	Decision Tree (DT), tailored to longitudinal real-time data	Spanakis et al., 2017
	Evaluate, how healthy Brazilian children and teens respond inter-individually to nutritional intervention of multivitamins and minerals, to develop recommendations for optimizing the levels of these supplements.	Elastic Net (EN) penalized regression model	Mathias et al., 2018
	Investigate whether the consideration of additional variables (in total 473 available variables, including dietary and nutritional information) could increase the accuracy of CVD risk prediction in 423,604 UK Biobank participants.	AutoPrognosis	Alaa et al., 2019
	Investigate whether the consideration of dietary information can improve CVD risk prediction.	Gradient Boosted Machines (GBMs) and Random Forests (RF), tailored to the analyses of survival data	Rigdon and Basu, 2019
**Gut microbiome**	Assess the potential of the (mainly gut) microbiome species-level abundances to be used for the classification of healthy vs. unhealthy (including obese and T2D patients) individuals.	Random Forests (RF), Support Vector Machine (SVM)	Pasolli et al., 2016
	Predict different traits, including cholesterol levels and BMI using the gut microbiome data in healthy participants.	Regularization of Learning Networks (RLN), Deep Neural Networks (DNNs), Gradient Boosting Trees (GBTs), Linear Models (LM)	Ira Shavitt, 2018
	Compare the composition of the gut microbiome in CAD patients vs. healthy controls.	Random Forests (RF)	Zhu et al., 2018
	Test, whether gut microbiome could be potentially used for diagnostic screening of CVD.	Random Forests (RF), Support Vector Machine (SVM), Decision Trees (DT), Elastic-Net (EN) and Neural Networks (NN)	Aryal et al., 2020
**Dietary factors and gut microbiome**	Identify associations between the gut microbiome composition and the concentration of butyrate, in response to dietary supplementation with resistant starch.	Random Forests (RF)	Venkataraman et al., 2016
	Investigate, the post-meal glucose levels in response to 46,898 standardized and real-life meals, in conjunction with the gut microbiome composition.	Stochastic Gradient Boosting Regression (SGBR)	Zeevi et al., 2015
	To validate the predictions by Zeevi et al. (2015) in an independent 327 cohort of individuals.	Stochastic Gradient Boosting Regression (SGBR)	Mendes-Soares et al., 2019
	Develop standardized protocols for the analyses of the diet-induced gut microbiome changes.		Spector et al., 2019
	Compare the post-meal glucose levels in response to the traditionally made sourdough-leavened whole-grain bread vs. industrially made white bread, in conjunction with the gut microbiome composition.	Stochastic Gradient Boosting Regression (SGBR)	Korem et al., 2017
	Use the gut microbiome data to predict changes of TMAO in healthy individuals after choline intake or screening population at high risks of CVD.	Random Forests (RF)	Lu et al., 2017

*Considering that only a few studies so far have used dietary factors (Alaa et al., 2019; Rigdon and Basu, 2019) or gut microbiome (Zhu et al., 2018; Aryal et al., 2020), and no studies using both (the closest being Zeevi et al., 2015 related to blood glucose levels), in combination with AI/ML for CAD risk prediction, we also consider closely related research on dietary factors (in green), gut microbiome (in blue) and combinations of both (in turquoise) in other disease settings vs. healthy individuals*.

## 2. Artificial Intelligence / Machine Learning Approaches for CAD Risk Prediction

Artificial Intelligence (AI) / Machine Learning (ML) has recently caught the interest of both academia and industry, and the different approaches have been explicitly reviewed elsewhere, e.g., Cao et al., 2018; Goecks et al., 2020. Hence, we only give a very brief overview, highlighting some of the most popular and widely used approaches and common terminology in the field, to prepare the reader for the sections to follow.

In general, AI/ML-based approaches can be considered as a set of methods that can effectively use large and complex data sets to extract meaningful patterns (i.e., “learn”) in order to use this “knowledge” to make predictions on other data (Vilne et al., 2019) and improve with experience (Libbrecht and Noble, 2015). Moreover AI/ML can be performed either (1) in a unsupervised manner by exploring and detecting what types of labels best explain the data i.e., using unlabeled data; (2) in a supervised manner by classifying, predicting and explaining the data, requiring labels (Vilne et al., 2019; Eetemadi et al., 2020), or (3) in a semi-supervised manner, taking advantage of both unlabeled and labeled data, where only a subset of data is labeled (Libbrecht and Noble, 2015).

In particular, supervised learning has gained much attention recently (Reel et al., 2021), as it allows to define certain outputs that can be used for classification of patients, and will be the main focus of this Mini-Review. In this class, one of the most popular is the Random Forest (RF) approach (Breiman, 2001), which randomly selects a subset from the training data to construct an ensemble of Decision Tree (DT) predictors to aggregate the predictions, by this attempting to lower the variance and deal with the issue of overfitting. Decision Tree (DT) approach is also a commonly used classifier, splitting the input data into branch-like segments, according to a certain parameter (Goecks et al., 2020).

Another popular method in the field is the Support Vector Machine (SVM) classifier, representing a pattern classification technique, based on the idea of transforming the original data that is not linearly separable to a higher dimensional space and finding a hyperplane separating the data into classes, based on a priori defined criteria, with the aim to overcome overfitting (Suykens et al., 2001). However, further improvements may be necessary when dealing with omics data (Han and Jiang, 2014).

Gradient Boosting (GB), such as Stochastic Gradient Boosting Regression (Friedman, 2001) is a technique that, similar to RF, constructs multiple decision trees by drawing a random samples from the data set (termed bagging). However, instead of constructing many parallel deep trees, it constructs multiple shallow trees (weak learners) and in a sequential manner (i.e., one after the other) so that the next tree improves upon the classification of previous trees in an additive manner. GB is known to perform best with fewer input variables of low dimensionality, whereas RF performs better with many input variables or high dimensionality (Hughes et al., 2019).

Finally, Artificial Neural Networks (ANN), and their extension, Deep Learning (DL), are graph computing models, which, at least to some extent, should mimic the functioning of the human brain, hence their computing units are called neurons and are interconnected for passing information to each other. Moreover, networks of neurons are additionally organized in layers. The first one is an input layer, receiving the training data. This is followed by several hidden layers. The last one is an output layer, which performs the actual prediction of the class (McCulloch and Pitts, 1990). ANN have been demonstrated to outperform other AI/ML approaches in many areas, especially (medical) image analyses (Eetemadi et al., 2020).

Performance of an AI/ML classifier is often expressed as the area under the curve (AUC), where a value of 0.5 indicates poor performance (equal to a random guess) while higher values (approaching 1) indicate better classification performance, allowing an easy comparison of the success of various implementations of AI/ML approaches (Bradley, 1997). However, considering that, in most cases, the users are more interested in positive outputs (i.e., people at high CAD risk), some other performance measures would need to be considered as well, such as the Jaccard index (J) or the F1-score, focusing on the fraction of true positives (Jiao and Du, 2016). Moreover, if the input data sets are imbalanced (i.e., many more controls than CAD patients in the training set), precision-recall (PR) curve should be considered along the ROC curve and additional performance measures, such as the balanced accuracy (BAcc) and the Matthew's Correlation Coefficient (MCC) considered (Jiao and Du, 2016). We refer the interested reader to Jiao and Du (2016) for more details. Moreover, if the input data is not normally distributed, maximum likelihood estimation (MLE) should be used to model this data and determine the model parameters for the evaluation metrics (Maximum-likelihood method, 2001).

The added value of AI/ML models in CAD diagnostics has been thoroughly reviewed before, examining 149 relevant studies between 1992 and 2019 (Alizadehsani et al., 2019). Most of this research focused on the usage of clinical data (symptom, examination and echo features), laboratory measurements and medical images (e.g., coronary computed tomography angiography, myocardial perfusion imaging or intravascular ultrasound) (Alizadehsani et al., 2019). The Authors observed that there were three approaches applied to almost all the datasets—ANN/DL, DT, and SVM—most probably due to their ease of use, low computational burden and encouraging performance (Alizadehsani et al., 2019). In particular, studies with best performances (i.e., with a reported accuracy of >98%) used ANN and SVM as their classifiers, which may be due to the use of non-linear kernel functions (Alizadehsani et al., 2019). However, it was concluded that further investigation are needed to determine which approaches are most appropriate for a particular feature category (e.g., ejection fraction, regional wall motion abnormality, and valvular heart disease extracted from echo). Of note, however, neither of the data types highlighted above provide any information on the molecular bases of a disease, which could possibly yield a more timely and precise diagnosis, or even risk prediction, allowing for individually tailored treatments (Westerlund et al., 2021) or even prevention strategies, toward the goal of “positive health”, resulting in a significantly improved life-span and quality (Movsisyan et al., 2020).

Genomic data have been used in combination with AI/ML for CAD risk prediction. In particular, (penalized) logistic regression, Naïve Bayes (NB), RF, SVM, and GB were compared vs. polygenic risk scores (PRS) on a data set of 7,736 CAD cases vs. 6,774 controls, testing the final models on an independent data set (527 CAD cases vs. 473 controls) (Gola et al., 2020). Interestingly, they found that PRS actually outperformed AI/ML-based approaches in predicting CAD status (AUC~0.92 vs. ~0.81 for NB and SVM and AUC~0.75 for RF and GB). The Authors conclude that “there is no need to use a sledge-hammer to crack the nut”, i.e., the assumption of linear additive effects influencing the risk of CAD seems sufficient. On the other hand, PRS may not be a suitable option, if the goal would be to predict the changes in CAD risk over time or the particular molecular basis driving the development and progression CAD (Westerlund et al., 2021).

This is were additional data layers such dietary factors and gut microbiome, as an integrator of this information (Bashiardes et al., 2018; Eetemadi et al., 2020), come in. However, only a few studies so far have used dietary factors (Alaa et al., 2019; Rigdon and Basu, 2019) or gut microbiome (Zhu et al., 2018; Aryal et al., 2020), and no studies using both (the closest being Zeevi et al., 2015 related to blood glucose levels), in combination with AI/ML for CAD risk prediction. We further discuss these few studies and also consider closely related research on dietary factors, gut microbiome and combinations of both in other disease settings vs. healthy individuals.

## 3. Performing Diet-Based CAD Risk Prediction Using AI/ML

Dietary information is mainly collected *via* questionnaires, either through self-reporting or by a trained interviewer. For self-reporting, a food frequency questionnaire and dietary recall can be used, where participants report their meal intake either every 24 h or over a longer period through a checklist of food items (Eetemadi et al., 2020). At the same time, fitness apps are gaining increased popularity, as food logging can be performed during its consumption or even by capturing an image of the meal, thus the bias related to individual's memory can be reduced (Weber and Achananuparp, 2016; Verma et al., 2018; Eetemadi et al., 2020). Clearly, such food tracking would be of utmost importance for a more efficient management of patients with obesity, T2D and CVD/CAD (Bernstein et al., 2010; Pallazola et al., 2019), when successfully coupled with an effective coaching to modulate it toward healthy food choices (Spanakis et al., 2017). The AI/ML approaches can be leveraged for such purposes (Verma et al., 2018).

In this regard, Myers et al. (2015) created a Google app, Im2Calories, to predict the nutritional contents and calories of individual's meal from its image, using a Convolutional Neural Network/DL-based classifier, which was modified to run on a mobile phone analyzing images taken by users, demonstrating promising first results in this direction.

Weber and Achananuparp (2016) used public food diaries of >4,000 MyFitnessPal users to train a SVM classifier to distinguish between a successful (“below” a user specified “daily calories goal”) vs. un-successful (“above” the goal) diet and analyzed the different dietary factors influencing these two outcomes. It was observed that “oil”, “butter”, “mcdonalds”, “dessert” or “pork” vs. “poultry” were related to being “above” the calories goal. Moreover, there were less food logging on the weekend and the users were most likely to be “above” the calories goal (Weber and Achananuparp, 2016).

Spanakis et al. (2017) made use of data collected from the fitness app ThinkSlim, to link the individual states (like location, activity, emotions- cheerful, relaxed vs. sad, bored, stressed, angry, worried) of healthy-weight vs. overweight individuals to their dietary choices or wishes, using a Decision Tree (DT)-based classifier, modified to use longitudinal real-time data. They derived several groups of individuals with similar eating behavior and used this information to warn the participants before the individual states that may lead to unhealthy eating behavior (Spanakis et al., 2017).

Mathias et al. (2018) conducted a six-week study to evaluate, how 136 healthy Brazilian children and teens (9–13 years old) responded to multivitamins and minerals, to develop recommendations for optimizing their levels, based on several clinical, anthropometric and food intake parameters. These data were then used to predict each individual's response to the intervention, based on these measures using an Elastic Net penalized regression model.

However, none of the above mentioned studies were directly related to CAD risk prediction. There have been only a few studies considering the dietary factors for CAD risk prediction, so far. Alaa et al. (2019) analyzed 423,604 UK Biobank participants without CVD at baseline with the aim to predict their future disease risk. They investigated, whether AI/ML-based approaches could possibly improve disease risk prediction, as compared to conventional approaches (such as FRS) and whether considering additional information (i.e., 473 variables, including dietary information) could increase the accuracy of their predictions. They used AutoPrognosis, which allows to automatically select and tune the best possible AI/ML approaches, by comprising different imputation strategies, feature selection and processing, as well as classification and calibration approaches. They observed and improvement in comparison to (AUC~0.77 vs. ~0.72 for FRS) conventional approaches (Alaa et al., 2019).

Rigdon and Basu (2019) performed a retrospective study using AI/ML exploring whether considering randomly sampled sparse nutrition data could possibly improve CVD mortality risk prediction. They made use of NHANES interview data collected from 1999 to 2011 linked to the National Death Index (NDI) in the US, selecting 29,390 participants as their training set and further 12,600 participants as their test set. Similarly to Alaa et al. (2019), they aimed at testing whether AI/ML-based approaches vs. standard (Cox) models and considering additional predictor variables (dietary information) could possibly improve CVD mortality risk prediction. They applied two DT-based AI/ML approaches the Gradient Boosted Machines (GBM) (Chen et al., 2013) and RF (Ishwaran et al., 2008), tailored to the analyses of survival data to demonstrated that the inclusion of dietary information significantly improved risk prediction, as compared to the standard models and when including only the traditional risk factors. In particular, they found that a standard Cox model without dietary factors overestimated the CVD mortality risk nearly two-fold, whereas AI/ML models in combination with these additional data substantially improved their predictions (AUC~0.87 vs. ~0.93).

## 4. Performing Gut Microbiome-Based CAD Risk Prediction Using AI/ML

In addition to genetic and environmental/life-style factors, gut microbiota has emerged as a additional factor influencing the CAD risk (Aryal et al., 2020). Clearly, researchers have asked, whether gut microbiome profiling combined with AI/ML approaches could be used for improved CAD/CVD risk prediction. In the last 10 years, a number of studies have demonstrated that there is a possible relationship between the gut microbiome composition, such as changes in the abundance of *Bacteroidetes, Firmicutes, Lactobacillus, Streptococcus, Bifidobacterium, Roseburia*, or *Escherichia* spp. and the development of several diseases, including obesity (Turnbaugh et al., 2009; Maruvada et al., 2017; Miyamoto et al., 2019) hypertension (Karbach et al., 2016), and CVD (Karlsson et al., 2012; Koeth et al., 2013; Miele et al., 2015; Kelly et al., 2016; Tang et al., 2017; Ascher and Reinhardt, 2018).

Several studies have used AI/ML approaches to classify test subjects into groups (such as healthy vs. disease) based on microbiome data. In most studies, relative abundances of microbiome taxa are used as features, obtained either by amplicon sequencing of the 16S rRNA phylogenetic marker gene or by shotgun metagenomic sequencing (Hughes et al., 2019). As the costs of shotgun metagenomic sequencing decrease, the functional profiles derived from metagenome sequences can be expected to increasingly be used as input features with AI/ML approaches (Eetemadi et al., 2020; Sanchez-Rodriguez et al., 2020). Pasolli et al. (2016) utilized 2,424 shotgun metagenomic samples from eight studies to assess the potential of the (mainly gut) microbiome species-level abundances to be used in order to differentiate healthy vs. unhealthy (including obese and T2D) individuals and compare the prediction accuracy of RF vs. SVM approaches. Interestingly, for T2D and obesity, the models demonstrated lower discrimination ability as compared, for example, to liver cirrhosis (AUC of 0.74 and 0.65 vs. 0.94, respectively), suggesting less significant changes in microbiome composition related to T2D and obesity. Comparing the accuracy of RF vs. SVM, in all cases, RF demonstrated similar or even better results than SVM (for T2D: AUC~0.74 vs. ~0.66, respectively).

However, although the approach of using gut microbiome in combination with AI/ML approaches for disease risk prediction is not novel, it has not been widely applied for CAD, yet (Aryal et al., 2020). Zhu et al. (2018) compared the composition of the gut microbiome between 70 CAD patients vs. 98 healthy controls and used RF to potentially differentiate these two groups of individuals, achieving and AUC of 0.67. In addition, the gut microbiome of CAD patients displayed decreased diversity and richness, with decreased abundances of *Faecalibacterium, Roseburia*, and *Eubacterium rectale* (the butyrate producers) and increased abundances of *Escherichia-Shigella* and *Enterococcus*. More recently, in order to test whether gut microbiome could be potentially used for diagnostic screening of CVD, Aryal et al. (2020) applied five different AI/ML approaches (RF, SVM, DT, Elastic-Net and Neural Networks) to the gut microbiome relative abundances of 478 CVD patients vs. 473 healthy controls, collected as part of the American Gut Project [https://microsetta.ucsd.edu/american-gut-project/] and profiled using fecal 16S ribosomal RNA sequencing. However, when using 39 differential bacterial taxa as features, the best AUC this study could achieve was AUC~0.58 (with Neural Networks), followed by Elastic-Net (AUC~0.57), SVM (AUC~0.55) and DT (AUC~0.51). Interestingly, the performance of RF significantly improved (AUC~0.65) when trained with the top 500 high-variance OTU features, instead of taxonomic features, whereas the AUC of Neural Networks dropped (AUC~0.48). Furthermore, highly contributing OTU features (HCOFs) were selected based on their variable importance (0–100, where 0: no contribution to the model and 100: max contribution to the model) to further reduce the dimensionality of the OTU feature space. The top 100 HCOFs with the highest scores were selected for training the RF model. As a result, the RF models trained with the top 20 and top 25 HCOFs achieved further improved performance (AUC~0.70).

## 5. Considering Diet-Gut Microbiome Interactions for CAD Risk Prediction Using AI/ML

It can be assumed that particular diets, such as those high in fats and/or sugars might lead to variations in the gut microbiome composition and changes in its functional capacity that potentially might facilitate the development of diseases, including metabolic disorders such as obesity, insulin resistance and atherosclerosis/CVD (Sanchez-Rodriguez et al., 2020). Despite the close link between our diet and gut microbiome, the number of studies collecting and analyzing both types of data is sparse and either not considering the full spectrum of dietary factors (Lu et al., 2017) or not directly addressing the prediction of CVD/CAD risk (Zeevi et al., 2015; Venkataraman et al., 2016; Spector et al., 2019).

Zeevi et al. (2015) used a the GB approach to investigate whether individuals' gut microbiome profiles in combination with several other sources of information (blood parameters, anthropometrics, self-reported lifestyle behaviors and physical activity) could predict glucose levels in response to standardized and real-life meals in a cohort of 800 overweight or obese non-diabetic individuals, observing high inter-individual variability, even in response to identical meals, suggesting that dietary recommendations need to be personalized. Later, these predictions were validated by Mendes-Soares et al. (2019) in an independent cohort of 327 individuals and by Korem et al. (2017), when focusing on the consumption of sourdough-leavened whole-grain bread vs. industrially made white bread, also using the GB approach. In the latter case, the relative abundances of *Coprobacter fastidiosus* and *Lachnospiraceae bacterium* were among the most informative features.

Venkataraman et al. (2016) used RF to predict whether the gut microbiome composition of individuals can predict their response to dietary supplementation with resistant starch, as measured using fecal butyrate concentrations. This study could identify three different response groups—enhanced, high and low, and could attribute these differences to the increase of starch-degrading bacteria *Bifidobacterium adolescentis* and *Ruminococcus bromii* in the enhanced and high, but not in the low fecal butyrate concentration group.

Spector et al. (2019), as part of the PREDICT study [http://www.tim-spector.co.uk/predict/], is actively working toward personalized nutrition tools by systematically analyzing the diet-induced gut microbiome changes using AI/ML approaches in order to stratify individual responses to dietary interventions based on the individual's gut microbiome and develop standardized protocols for the purpose. Among others, this study has demonstrated that shotgun metagenomic sequencing may be more accurate than 16S rRNA amplicon sequencing, as it allows also capturing individual-specific strain-level features, thus improving the stratification.

In the context of CVD/CAD risk prediction, most studies have focused on the circulating levels of the diet- and gut microbiota-dependent metabolite trimethylamine-N-oxide (TMAO) (Trøseid et al., 2020). Lu et al. (2017) used RF and the gut microbiome data to predict changes of TMAO levels after choline intake, as a potential approach for screening population at high risk of CVD and identified the beta (inter-individual) diversity of the gut microbiome as a significant predictor (AUC of 0.86) of increased vs. decreased plasma TMAO level.

## 6. Discussion

Timely and precise identification of people at high risk of CAD is of utmost importance for the development of personalized treatment strategies (Alaa et al., 2019; Westerlund et al., 2021), as such persons may need more aggressive health promotion strategies, especially the modifiable CAD risk factors could be effectively reduced or even eliminated in this way (Movsisyan et al., 2020). Although, numerous algorithms for CAD risk prediction have been developed over the years and several have also entered the clinical routine (FRS Wilson et al., 1998, SCORE Conroy et al., 2003), these are typically based on a limited number of traditional CAD risk factors (age, sex, diabetes, LDL and HDL cholesterol, smoking, systolic blood pressure) and are not suitable across all patient groups (Alaa et al., 2019) and do not take into account the fact that by modifying the person's environment/lifestyle the disease risk could be reduced over time (Westerlund et al., 2021).

The added value of AI/ML approaches in CAD diagnostics has been explored before, however, so far, most of this research has focused on the usage of clinical data and medical images (Alizadehsani et al., 2019), thus providing no information on the molecular bases of a disease (Westerlund et al., 2021). A small number of studies has used genomic data have been used in combination with AI/ML for CAD risk prediction (Gola et al., 2020). However, AI/ML approaches underperformed in comparison to a simple PRS, assuming linear additive effects (Gola et al., 2020). This is were additional data layers such dietary factors and gut microbiome, as an integrator of this information (Bashiardes et al., 2018; Eetemadi et al., 2020), come in. However, only a few studies so far have used dietary factors (Alaa et al., 2019; Rigdon and Basu, 2019) or gut microbiome (Zhu et al., 2018; Aryal et al., 2020), and no studies using both [the closest being (Zeevi et al., 2015) related to blood glucose levels], in combination with AI/ML for CAD risk prediction. We further discuss these few studies and also consider closely related research on dietary factors, gut micorbiome and combinations of both in other disease settings vs. healthy individuals.

With the advent of wearable biosensors connected to mobile applications, large-scale longitudinal food diaries and images of meals consumed will become increasingly available providing a valuable data source for such investigations (Munos et al., 2016). The future vision for personalized nutrition has led to great interest for advancements in the diagnostics and decision support systems (DSS) that would allow continuous assessment of individual's dietary features, in conjunction with gut microbiome composition and additional information, such as access to the electronic health record (EHR) and lifestyle and environment information, physical activity from the biosensors and wearable health technology. All of it would aid in forming tailored recommendations such as choosing an optimal meal for lowering post-meal glucose levels (as shown by Zeevi et al., 2015) in patients with T2D. Although, the recent re-emergence of AI/ML approaches is opening intriguing perspectives in this direction, it must be remembered that these data-driven technologies and their predictions strongly depend on the quantity and quality of the input data. In this regard, several limitations to the current food intake and composition databases have been observed. Apparently, these databases currently contain only 0.5% of the known nutritional compounds (Eetemadi et al., 2020). Another issue is the data standardization, which is challenging as complex dietary patterns need to be captured in an organized manner, translating chemicals constituents of the food into the intake of energy and nutrients (Verma et al., 2018). Currently, the most widely applied methods of food intake monitoring include the food diaries, which make it difficult to convert the food descriptions into the energy. Additional challenges arise when the food is collected from different sources, i.e., individual and/or hospital-based sources. The missing data problem could be partly addressed through improved data imputation techniques, which should be complemented by improved food intake monitoring and data collection methods, creating integrated databases with defined standard formats for annotation and classification, considering the FAIR (Findability, Accessibility, Interoperability, and Reuse) data principles [https://www.go-fair.org/fair-principles/]. Initiatives such as the EuroDISH project [https://www.eurofir.org/our-resources/past-projects/eurodish/] are already working in this direction.

There are a number of challenges and limitations related to the application of AI/ML approaches for microbiome studies, as thoroughly and systematically summarized in several literature reviews (Marcos-Zambrano et al., 2021; Moreno-Indias et al., 2021) by the members of the COST Action CA18131 “ML4Microbiome” (https://www.cost.eu/actions/CA18131/), bringing together AI/ML experts and microbiome researchers. Overall, similar to other high-throughput studies, one of the main limitations in current research has been the usage of inappropriate study design, including small datasets and lack of additional data to estimate confounding effects, especially considering the well-known huge variations in microbiome composition across individuals and body sites and their strong dependence on the environment/lifestyle factors such as geographic location, diet and medications (Marcos-Zambrano et al., 2021; Moreno-Indias et al., 2021). In order to identify generalized responses, a much larger number of individuals spanning a range of microbiome types and a careful adjustment for potential confounding effects would be required (Johnson et al., 2020). In addition, a number of data processing/statistical and AI/ML challenges have been observed, such as the selection of appropriate normalization methods to address the variability in raw read counts, inappropriate distributional assumptions considering the data sparsity, compositional nature and complex and hierarchical dependency structures, the choice of suitable feature selection approaches, i.e., requiring customized analytical approaches (Eetemadi et al., 2020; Marcos-Zambrano et al., 2021; Moreno-Indias et al., 2021). In fact, successful examples often present a combination of different statistical approaches, specifically tailored to the characteristics of different data types (Marcos-Zambrano et al., 2021; Moreno-Indias et al., 2021). On top of that the dependence on the reference databases is a well-known major limitation of the sequence alignment-based approaches, used to assign taxa in sequencing studies (Chaudhary et al., 2015; Vilne et al., 2019), resulting in large numbers of uncharacterized microbes (the “microbial dark matter”) (Marcos-Zambrano et al., 2021). Finally, the field of high-throughput sequencing overall needs a rigorous assessment, benchmarking and standardization of approaches and tools (Vilne et al., 2019), to allow cross-study comparisons and modeling (Marcos-Zambrano et al., 2021). Currently, the integration of microbiome data across several studies is difficult due to the above mentioned factors, as well as the differences in sample collection, storage and processing protocols in the wet-lab, which may introduce biases (Eetemadi et al., 2020). Hence, all findings should be validated, e.g., using quantitative PCR (Jian et al., 2020). Finally, also for these data, the above mentioned FAIR data principles should be widely incorporated [https://www.go-fair.org/fair-principles/] to facilitate such efforts. For more details we refer the reader to Marcos-Zambrano et al. (2021); Moreno-Indias et al. (2021). However, we note that these current limitations related to microbiome studies are posing additional challenges for CAD risk prediction.

Moreover, several studies have shown that the inter-individual responses to dietary factors may differ, mostly due to the differences in the gut microbiome composition (Zeevi et al., 2015; Korem et al., 2017; Mendes-Soares et al., 2019). However, especially in the context of multi-factorial diseases, such as CAD, the differences in individual genetic predisposition (Nikpay et al., 2015; Nelson et al., 2017) and its down-stream implications (Brænne et al., 2015; Vilne et al., 2017; Lempiäinen et al., 2018; Vilne and Schunkert, 2018) in addition to variations in other (besides diet) environmental and lifestyle factors such as physical activity, stress and sleep may play and important role in these responses (Khera et al., 2017). Hence, emphasizing the need to collect a wide variety of measures in large populations that would allow for stratification of patients in sub-groups and perform longitudinal sampling to also capture the dynamics of these responses. Endeavors to standardize the study protocols have already started (Spector et al., 2019).

On the other hand, benchmark investigations have demonstrated that, whether a particular AI/ML approach would actually improve the predictions compared to conventional approaches, may depended on the specific dataset at hand (Westerlund et al., 2021). For example, in microbiome studies, DL approaches have been demonstrated to underperform in comparison to GB, possibly due to the potentially large variability in the relative importance of different input features. To overcome this limitation, Ira Shavitt (2018) have proposed an approach called Regularization of Learning Networks (RLN). They used it to predict a number of traits related to disease risk, such as cholesterol levels and body mass index (BMI) from the gut microbiome data of 2,574 healthy individuals. They evaluated four different AI/ML approaches [RLN, GB, DL, and Linear Models (LM)] and, although, GB still outperformed the other three, RLN performed significantly better than DL (15% vs. ca. 2% less explained variance than GB on average).

Currently, the number of studies investigating the potential of gut-microbiome in combination with AI/ML to predict CVD risk is limited (Aryal et al., 2020) an so is the prediction power of these models, with a max AUC of 0.70, when training a RF model with the top 25 highest contributing OTU features (Aryal et al., 2020). However, it must be noted that the authors did not normalize the OTU data across all the samples to test the option of classifying new samples without the need for repeated processing (Aryal et al., 2020). In addition, this study addressed the prediction of CVD, which, as the authors themselves recognize (Aryal et al., 2020) includes a range of conditions (from hypertension and atherosclerosis to CAD). Hence, these predictions may improve when stratifying CVD patients into specific disease sub-types. Moreover, another interesting observation from this study is the fact that bacterial taxonomic features achieved a lower (AUC~0.58) AUC, in comparison to high-variance OTU features (AUC~0.65), and especially when further reducing the dimensionality of the feature space by pre-selecting the top 25 highest contributing OTU features (AUC~0.70) (Aryal et al., 2020). From the usage in clinical routine, focusing on a small number of highly contributing OTUs may be indeed more practical, analogous to the handful of traditional CAD risk factors, however, we will need further studies to arrive replicate these findings and arrive at these gut microbiome biomarkers. Furthermore, their mechanistic implications in CVD need to be further investigated (Aryal et al., 2020). Gut microbiota as the only type of data used for diagnostic classification of non-CVD vs. CVD may not be sufficient Especially, considering that gut microbiome can be influenced by other features such as diet and medications, hence these data should be always collected in parallel.

Clearly, AI/ML (especially DL approaches due to their capabilities to learn from input raw data, instead of using hand-crafted features that require domain expertise, Ching et al., 2018) in combination with timely access to numerous, potentially relevant, data sources [e.g., gut microbiome and genetic data, in addition to the current 7 CVD metrics smoking, physical activity, body mass index, blood pressure, cholesterol, glucose and dietary factors (Angell et al., 2020), combined with longitudinal clinical data from electronic health records (Matlock et al., 2013; Reynolds et al., 2017)] also holds a great promise for the improvements of public health surveillance systems, formulation of policies by forecasting the impact of a factor or intervention on the burden of disease and the cost of care, and to propose recommendations to stakeholders (medical institutions, public health authorities, scientific communities) enabling public health action and measure progress with the aim to reduce the huge socio-economic burden of CVD/CAD and increase healthy life expectancy in future (Angell et al., 2020; Roger et al., 2020). The same is true for the implementation of personalized decision support system (DSS) for CAD risk prediction and patient management that would be a great support for clinicians in health care.

However, despite the rapid development of several technologies and advancements in Big Data analytics, the implementation of such systems that would integrate comprehensive health and related data (such as genetic variations, dietary factors, gut microbiome) to provide either generalized recommendations for public health surveillance and policy makers or individual recommendations for the routine clinical practice, still poses a number of challenges that will need to be overcame first, in order to move toward their implementation and usability in practice. Overall, such systems will need to deal with heterogeneous datasets and we will require a rigorous assessment, benchmarking and standardization of AI/ML-based CVD/CAD risk prediction models, ensuring model availability and extensive multiple external validations and calibration across different disease outcomes, populations (in men and women separately) and geographical regions *via* head-to-head comparisons across different studies and model impact and performance generalizability assessment and to identify potential sources of heterogeneity (Damen et al., 2016; Marcos-Zambrano et al., 2021; Westerlund et al., 2021).

Moreover, In April 2016, the European Union adopted new rules regarding the use of personal information, the General Data Protection Regulation, which imposes additional legal and privacy constraints when analyzing sensitive health data, hence model training will need to be accomplished within a differential privacy framework without sharing the raw data (e.g., federated learning) and considering other rules regarding the use of personal information as input for decision-making approaches, such as the ‘right to an explanation', meaning that when using AI/ML, we must be able to explain how a decision was reached, especially if the ground-truth is unknown (Ching et al., 2018). This calls for the AI/ML models to be human-interpretable, reliable and explainable to aid the formulation guidelines or personalized advice on treatment strategy, or even prevention, plans (Ching et al., 2018; Westerlund et al., 2021).

In any case, the AI/ML tools will not be a replacement for the human experts, who are still an integral part of the knowledge discovery process, hence, managing huge amounts of health data will need to become an integral part of future medical, policy making and research activity, across sub-disciplines (Moreira et al., 2019).

## Author Contributions

BV wrote the manuscript. JĶ, IS, IL, AK, and OV participated in revising and editing the manuscript. All authors have read and approved the final version of the manuscript.

## Funding

This research was funded by the Latvian Council of Science within the project Gut microbiome composition and diversity among health and lifestyle induced dietary regimen, project No. lzp-2018/2-0266.

## Conflict of Interest

The authors declare that the research was conducted in the absence of any commercial or financial relationships that could be construed as a potential conflict of interest.

## Publisher's Note

All claims expressed in this article are solely those of the authors and do not necessarily represent those of their affiliated organizations, or those of the publisher, the editors and the reviewers. Any product that may be evaluated in this article, or claim that may be made by its manufacturer, is not guaranteed or endorsed by the publisher.

## References

[B1] Aherrahrou R. Aherrahrou Z. Schunkert H. Erdmann J. (2017). Coronary artery disease associated gene phactr1 modulates severity of vascular calcification *in vitro*. Biochem. Biophys. Res. Commun. 491, 396–402. 10.1016/j.bbrc.2017.07.090 28720499

[B2] Alaa A. M. Bolton T. Di Angelantonio E. Rudd J. H. F. van der Schaar M. (2019). Cardiovascular disease risk prediction using automated machine learning: a prospective study of 423,604 UK biobank participants. PLoS ONE 14, e0213653. 10.1371/journal.pone.0213653 31091238 PMC6519796

[B3] Alizadehsani R. Abdar M. Roshanzamir M. Khosravi A. Kebria P. M. Khozeimeh F.. (2019). Machine learning-based coronary artery disease diagnosis: a comprehensive review. Comput. Biol. Med. 111, 103346. 10.1016/j.compbiomed.2019.103346 31288140

[B4] Angell S. Y. McConnell M. V. Anderson C. A. Bibbins-Domingo K. Boyle D. S. Capewell S.. (2020). The American heart association 2030 impact goal: a presidential advisory from the american heart association. Circulation 141, e120–e138. 10.1161/CIR.0000000000000758 31992057 PMC8690536

[B5] Aryal S. Alimadadi A. Manandhar I. Joe B. Cheng X. (2020). Machine learning strategy for gut microbiome-based diagnostic screening of cardiovascular disease. Hypertension 76, 1555–1562. 10.1161/HYPERTENSIONAHA.120.15885 32909848 PMC7577586

[B6] Ascher S. Reinhardt C. (2018). The gut microbiota: an emerging risk factor for cardiovascular and cerebrovascular disease. Eur. J. Immunol. 48, 564–575. 10.1002/eji.201646879 29230812

[B7] Bashiardes S. Godneva A. Elinav E. Segal E. (2018). Towards utilization of the human genome and microbiome for personalized nutrition. Curr. Opin. Biotechnol. 51, 57–63. 10.1016/j.copbio.2017.11.013 29223004

[B8] Bernstein A. M. Sun Q. Hu F. B. Stampfer M. J. Manson J. E. Willett W. C. (2010). Major dietary protein sources and risk of coronary heart disease in women. Circulation 122, 876–883. 10.1161/CIRCULATIONAHA.109.915165 20713902 PMC2946797

[B9] Bodnar L. M. Cartus A. R. Kirkpatrick S. I. Himes K. P. Kennedy E. H. Simhan H. N.. (2020). Machine learning as a strategy to account for dietary synergy: an illustration based on dietary intake and adverse pregnancy outcomes. Am. J. Clin. Nutr. 111, 1235–1243. 10.1093/ajcn/nqaa027 32108865 PMC7266693

[B10] Brænne I. Civelek M. Vilne B. Di Narzo A. Johnson A. D. Zhao Y.. (2015). Prediction of causal candidate genes in coronary artery disease loci. Arterioscler. Thromb. Vasc. Biol. 35, 2207–2217. 10.1161/ATVBAHA.115.306108 26293461 PMC4583353

[B11] Bradley A. P.. (1997). The use of the area under the roc curve in the evaluation of machine learning algorithms. Pattern Recogn. 30, 1145–1159. 10.1016/S0031-3203(96)00142-2

[B12] Breiman L.. (2001). Random forests. Mach. Learn. 45, 5–32. 10.1023/A:1010933404324

[B13] Cao C. Liu F. Tan H. Song D. Shu W. Li W.. (2018). Deep learning and its applications in biomedicine. Genomics Proteomics Bioinform. 16, 17–32. 10.1016/j.gpb.2017.07.003 29522900 PMC6000200

[B14] Cecile A. Janssens J. W. Joyner M. J. (2019). Polygenic risk scores that predict common diseases using millions of single nucleotide polymorphisms: is more, better? Clin. Chem. 65, 609–611. 10.1373/clinchem.2018.296103 30808642

[B15] Chaudhary N. Sharma A. K. Agarwal P. Gupta A. Sharma V. K. (2015). 16s classifier: a tool for fast and accurate taxonomic classification of 16s rrna hypervariable regions in metagenomic datasets. PLoS ONE 10, e0116106. 10.1371/journal.pone.0116106 25646627 PMC4315456

[B16] Chen Y. Jia Z. Mercola D. Xie X. (2013). A gradient boosting algorithm for survival analysis via direct optimization of concordance index. Comput. Math. Methods Med. 2013, 873595. 10.1155/2013/873595 24348746 PMC3853154

[B17] Ching T. Himmelstein D. S. Beaulieu-Jones B. K. Kalinin A. A. Do B. T. Way G. P.. (2018). Opportunities and obstacles for deep learning in biology and medicine. J. R. Soc. Interface 15, 20170387. 10.1098/rsif.2017.0387 29618526 PMC5938574

[B18] Conroy R. M. Pyorala K. Fitzgerald A. P. Sans S. Menotti A. De Backer G.. (2003). Estimation of ten-year risk of fatal cardiovascular disease in Europe: the score project. Eur. Heart J. 24, 987–1003. 10.1016/s0195-668x(03)00114-3 12788299

[B19] Damen J. A. A. G. Hooft L. Schuit E. Debray T. P. A. Collins G. S. Tzoulaki I.. (2016). Prediction models for cardiovascular disease risk in the general population: systematic review. BMJ 353, i2416. 10.1136/bmj.i2416 27184143 PMC4868251

[B20] Davey Smith G. Ebrahim S. Lewis S. Hansell A. L. Palmer L. J. Burton P. R. (2005). Genetic epidemiology and public health: hope, hype, and future prospects. Lancet 366, 1484–1498. 10.1016/S0140-6736(05)67601-5 16243094

[B21] De Filippis F. Pellegrini N. Vannini L. Jeffery I. B. La Storia A. Laghi L.. (2016). High-level adherence to a mediterranean diet beneficially impacts the gut microbiota and associated metabolome. Gut 65, 1812–1821. 10.1136/gutjnl-2015-309957 26416813

[B22] Deloukas P. Kanoni S. Willenborg C. Farrall M. Assimes T. L. Thompson J. R.. (2012). Large-scale association analysis identifies new risk loci for coronary artery disease. Nat. Genet. 45, 25–33. 10.1038/ng.2480 23202125 PMC3679547

[B23] Dimovski K. Orho-Melander M. Drake I. (2019). A favorable lifestyle lowers the risk of coronary artery disease consistently across strata of non-modifiable risk factors in a population-based cohort. BMC Public Health 19, 1575. 10.1186/s12889-019-7948-x 31775698 PMC6882082

[B24] Dinh-Le C. Chuang R. Chokshi S. Mann D. (2019). Wearable health technology and electronic health record integration: scoping review and future directions. JMIR mHealth uHealth 7, e12861. 10.2196/12861 31512582 PMC6746089

[B25] Eetemadi A. Rai N. Pereira B. M. P. Kim M. Schmitz H. Tagkopoulos I. (2020). The computational diet: a review of computational methods across diet, microbiome, and health. Front. Microbiol. 11, 393. 10.3389/fmicb.2020.00393 32318028 PMC7146706

[B26] Erdmann J. Grosshennig A. Braund P. S. Konig I. R. Hengstenberg C. Hall A. S.. (2009). New susceptibility locus for coronary artery disease on chromosome 3q22.3. Nat. Genet. 41, 280–282. 10.1038/ng.307 19198612 PMC2695543

[B27] Erdmann J. Kessler T. Munoz Venegas L. Schunkert H. (2018). A decade of genome-wide association studies for coronary artery disease: the challenges ahead. Cardiovasc. Res. 114, 1241–1257. 10.1093/cvr/cvy084 29617720

[B28] Friedman J. H.. (2001). Greedy function approximation: a gradient boosting machine. Ann. Statist. 29, 1189–1232. 10.1214/aos/1013203451

[B29] Garud N. R. Pollard K. S. (2020). Population genetics in the human microbiome. Trends Genet. 36, 53–67. 10.1016/j.tig.2019.10.010 31780057

[B30] Goecks J. Jalili V. Heiser L. M. Gray J. W. (2020). How machine learning will transform biomedicine. Cell 181, 92–101. 10.1016/j.cell.2020.03.022 32243801 PMC7141410

[B31] Gola D. Erdmann J. Muller-Myhsok B. Schunkert H. Konig I. R. (2020). Polygenic risk scores outperform machine learning methods in predicting coronary artery disease status. Genet. Epidemiol. 44, 125–138. 10.1002/gepi.22279 31922285

[B32] Han H. Jiang X. (2014). Overcome support vector machine diagnosis overfitting. Cancer Inform. 13(Suppl 1), 145–158. 10.4137/CIN.S13875 25574125 PMC4264614

[B33] Ho F. K. Gray S. R. Welsh P. Petermann-Rocha F. Foster H. Waddell H.. (2020). Associations of fat and carbohydrate intake with cardiovascular disease and mortality: prospective cohort study of uk biobank participants. BMJ 368, m688. 10.1136/bmj.m688 32188587 PMC7190059

[B34] Howson J. M. M. Zhao W. Barnes D. R. Ho W.-K. Young R. Paul D. S.. (2017). Fifteen new risk loci for coronary artery disease highlight arterial-wall-specific mechanisms. Nat. Genet. 49, 1113–1119. 10.1038/ng.3874 28530674 PMC5555387

[B35] Hughes R. L. Marco M. L. Hughes J. P. Keim N. L. Kable M. E. (2019). The role of the gut microbiome in predicting response to diet and the development of precision nutrition models-part I: overview of current methods. Adv. Nutr. 10, 953–978. 10.1093/advances/nmz022 31225589 PMC6855943

[B36] Inouye M. Abraham G. Nelson C. P. Wood A. M. Sweeting M. J. Dudbridge F.. (2018). Genomic risk prediction of coronary artery disease in 480,000 adults: Implications for primary prevention. J. Am. Coll. Cardiol. 72, 1883–1893. 10.1016/j.jacc.2018.07.079 30309464 PMC6176870

[B37] Ira Shavitt E. S.. (2018). “Regularization learning networks: deep learning for tabular datasets,” in 32nd Conference on Neural Information Processing Systems (NeurIPS 2018) (Montreal, QC).

[B38] Ishwaran Udaya, B. Kogalur E. H. B. Lauer M. S. (2008). Random survival forests. Ann. Appl. Stat. 2, 841–860. 10.1214/08-AOAS169

[B39] Jian C. Luukkonen P. Yki-Jarvinen H. Salonen A. Korpela K. (2020). Quantitative PCR provides a simple and accessible method for quantitative microbiota profiling. PLoS ONE 15, e0227285. 10.1371/journal.pone.0227285 31940382 PMC6961887

[B40] Jiao Y. Du P. (2016). Performance measures in evaluating machine learning based bioinformatics predictors for classifications. Quant. Biol. 4, 320–330. 10.1007/s40484-016-0081-2

[B41] Johnson A. J. Zheng J. J. Kang J. W. Saboe A. Knights D. Zivkovic A. M. (2020). A guide to diet-microbiome study design. Front. Nutr. 7, 79. 10.3389/fnut.2020.00079 32596250 PMC7303276

[B42] Karbach S. H. Schonfelder T. Brandao I. Wilms E. Hormann N. Jackel S.. (2016). Gut microbiota promote angiotensin ii-induced arterial hypertension and vascular dysfunction. J. Am. Heart Assoc. 5, e003698. 10.1161/JAHA.116.003698 27577581 PMC5079031

[B43] Karlsson F. H. Fak F. Nookaew I. Tremaroli V. Fagerberg B. Petranovic D.. (2012). Symptomatic atherosclerosis is associated with an altered gut metagenome. Nat. Commun. 3, 1245. 10.1038/ncomms2266 23212374 PMC3538954

[B44] Kelly T. N. Bazzano L. A. Ajami N. J. He H. Zhao J. Petrosino J. F.. (2016). Gut microbiome associates with lifetime cardiovascular disease risk profile among bogalusa heart study participants. Circ. Res. 119, 956–964. 10.1161/CIRCRESAHA.116.309219 27507222 PMC5045790

[B45] Kessler T. Vilne B. Schunkert H. (2016). The impact of genome-wide association studies on the pathophysiology and therapy of cardiovascular disease. EMBO Mol. Med. 8, 688–701. 10.15252/emmm.201506174 27189168 PMC4931285

[B46] Kessler T. Wobst J. Wolf B. Eckhold J. Vilne B. Hollstein R.. (2017). Functional characterization of the, javax.xml.bind.jaxbelement@3a826464, coronary artery disease risk locus. Circulation 136, 476–489. 10.1161/CIRCULATIONAHA.116.024152 28487391 PMC5560301

[B47] Kessler T. Zhang L. Liu Z. Yin X. Huang Y. Wang Y.. (2015). Adamts-7 inhibits re-endothelialization of injured arteries and promotes vascular remodeling through cleavage of thrombospondin-1. Circulation 131, 1191–1201. 10.1161/CIRCULATIONAHA.114.014072 25712208

[B48] Khera A. V. Chaffin M. Aragam K. G. Haas M. E. Roselli C. Choi S. H.. (2018). Genome-wide polygenic scores for common diseases identify individuals with risk equivalent to monogenic mutations. Nat. Genet. 50, 1219–1224. 10.1038/s41588-018-0183-z 30104762 PMC6128408

[B49] Khera A. V. Emdin C. A. Kathiresan S. (2017). Genetic risk, lifestyle, and coronary artery disease. N. Engl. J. Med. 376, 1194–1195. 10.1056/NEJMc1700362 28328341

[B50] Koeth R. A. Wang Z. Levison B. S. Buffa J. A. Org E. Sheehy B. T.. (2013). Intestinal microbiota metabolism of l-carnitine, a nutrient in red meat, promotes atherosclerosis. Nat. Med. 19, 576–585. 10.1038/nm.3145 23563705 PMC3650111

[B51] Korem T. Zeevi D. Zmora N. Weissbrod O. Bar N. Lotan-Pompan M.. (2017). Bread affects clinical parameters and induces gut microbiome-associated personal glycemic responses. Cell Metab. 25:1243.e5–1253.e5. 10.1016/j.cmet.2017.05.002 28591632

[B52] Lempiäinen H. Brænne I. Michoel T. Tragante V. Vilne B. Webb T. R.. (2018). Network analysis of coronary artery disease risk genes elucidates disease mechanisms and druggable targets. Sci. Rep. 8, 3434. 10.1038/s41598-018-20721-6 29467471 PMC5821758

[B53] Libbrecht M. W. Noble W. S. (2015). Machine learning applications in genetics and genomics. Nat. Rev. Genet. 16, 321–332. 10.1038/nrg3920 25948244 PMC5204302

[B54] Lieb W. Vasan R. S. (2020). An update on genetic risk scores for coronary artery disease: are they useful for predicting disease risk and guiding clinical decisions? Expert Rev. Cardiovasc. Ther. 18, 443–447. 10.1080/14779072.2020.1797489 32672491

[B55] Lopez A. D. Mathers C. D. Ezzati M. Jamison D. T. Murray C. J. L. (2006). Global and regional burden of disease and risk factors, 2001: systematic analysis of population health data. Lancet 367, 1747–1757. 10.1596/978-0-8213-6262-4 16731270

[B56] Lu J.-Q. Wang S. Yin J. Wu S. He Y. Zheng H.-M.. (2017). [A machine learning model using gut microbiome data for predicting changes of trimethylamine-n-oxide in healthy volunteers after choline consumption]. J. Southern Med. Univ. 37, 290–295. 10.3969/j.issn.1673-4254.2017.03.02 28377341 PMC6780447

[B57] Marcos-Zambrano L. J. Karaduzovic-Hadziabdic K. Loncar Turukalo T. Przymus P. Trajkovik V. Aasmets O.. (2021). Applications of machine learning in human microbiome studies: a review on feature selection, biomarker identification, disease prediction and treatment. Front. Microbiol. 12, 634511. 10.3389/fmicb.2021.634511 33737920 PMC7962872

[B58] Maruvada P. Leone V. Kaplan L. M. Chang E. B. (2017). The human microbiome and obesity: moving beyond associations. Cell Host Microbe 22, 589–599. 10.1016/j.chom.2017.10.005 29120742

[B59] Mathias M. G. Coelho-Landell C. A. Scott-Boyer M.-P. Lacroix S. Morine M. J. Salomao R. C.. (2018). Clinical and vitamin response to a short-term multi-micronutrient intervention in Brazilian children and teens: from population data to interindividual responses. Mol. Nutr. Food Res. 62, e1700613. 10.1002/mnfr.201700613 29368422 PMC6120145

[B60] Matlock D. D. Groeneveld P. W. Sidney S. Shetterly S. Goodrich G. Glenn K.. (2013). Geographic variation in cardiovascular procedure use among medicare fee-for-service vs medicare advantage beneficiaries. JAMA 310, 155. 10.1001/jama.2013.7837 23839749 PMC4021020

[B61] Maximum-likelihood method. (2001). Encyclopedia of Mathematics (EMS Press). Available online at: https://encyclopediaofmath.org/wiki/Maximum-likelihood_method

[B62] McCulloch W. S. Pitts W. (1990). A logical calculus of the ideas immanent in nervous activity. Bull. Math. Biol. 52, 99–115; discussion: 73–97. 10.1016/S0092-8240(05)80006-0 2185863

[B63] Mendes-Soares H. Raveh-Sadka T. Azulay S. Edens K. Ben-Shlomo Y. Cohen Y.. (2019). Assessment of a personalized approach to predicting postprandial glycemic responses to food among individuals without diabetes. JAMA Netw. Open 2, e188102. 10.1001/jamanetworkopen.2018.8102 30735238 PMC6484621

[B64] Micha R. Wallace S. K. Mozaffarian D. (2010). Red and processed meat consumption and risk of incident coronary heart disease, stroke, and diabetes mellitus: a systematic review and meta-analysis. Circulation 121, 2271–2283. 10.1161/CIRCULATIONAHA.109.924977 20479151 PMC2885952

[B65] Miele L. Giorgio V. Alberelli M. A. De Candia E. Gasbarrini A. Grieco A. (2015). Impact of gut microbiota on obesity, diabetes, and cardiovascular disease risk. Curr. Cardiol. Rep. 17, 120. 10.1007/s11886-015-0671-z 26497040

[B66] Miyamoto J. Igarashi M. Watanabe K. Karaki S.-I. Mukouyama H. Kishino S.. (2019). Gut microbiota confers host resistance to obesity by metabolizing dietary polyunsaturated fatty acids. Nat. Commun. 10, 4007. 10.1038/s41467-019-11978-0 31488836 PMC6728375

[B67] Moraes Lopes M. H. B. Ferreira D. D. Ferreira A. C. B. H. da Silva G. R. Caetano A. S. Braz V. N. (2020). “Use of artificial intelligence in precision nutrition and fitness,” in Artificial Intelligence in Precision Health: From Concept to Applications, ed D. Barh (London; Cambridge; Oxford; San Diego, CA: Elsevier Science), 465–496. 10.1016/B978-0-12-817133-2.00020-3

[B68] Moreira M. W. L. Rodrigues J. J. P. C. Korotaev V. Al-Muhtadi J. Kumar N. (2019). A comprehensive review on smart decision support systems for health care. IEEE Systems Journal 13, 3536–3545. 10.1109/JSYST.2018.2890121

[B69] Moreno-Indias I. Lahti L. Nedyalkova M. Elbere I. Roshchupkin G. Adilovic M.. (2021). Statistical and machine learning techniques in human microbiome studies: contemporary challenges and solutions. Front. Microbiol. 12, 635781. 10.3389/fmicb.2021.635781 33692771 PMC7937616

[B70] Movsisyan N. K. Vinciguerra M. Medina-Inojosa J. R. Lopez-Jimenez F. (2020). Cardiovascular diseases in central and eastern Europe: a call for more surveillance and evidence-based health promotion. Ann. Glob. Health 86, 21. 10.5334/aogh.2713 32166066 PMC7059421

[B71] Munos B. Baker P. C. Bot B. M. Crouthamel M. de Vries G. Ferguson I.. (2016). Mobile health: the power of wearables, sensors, and apps to transform clinical trials. Ann. N. Y. Acad. Sci. 1375, 3–18. 10.1111/nyas.13117 27384501

[B72] Myers A. Johnston N. Rathod V. Korattikara A. Gorban A. Silberman N.. (2015). “Im2calories: towards an automated mobile vision food diary,” in IEEE International Conference on Computer Vision (ICCV) (Santiago). 10.1109/ICCV.2015.146

[B73] Neiburga K. Vilne B. Bauer S. Bongiovanni D. Ziegler T. Lachmann M.. (2021). Vascular tissue specific miRNA profiles reveal novel correlations with risk factors in coronary artery disease. Biomolecules 11, 1683. 10.3390/biom11111683 34827683 PMC8615466

[B74] Nelson C. P. Goel A. Butterworth A. S. Kanoni S. Webb T. R. Marouli E.. (2017). Association analyses based on false discovery rate implicate new loci for coronary artery disease. Nat. Genet. 49, 1385–1391. 10.1038/ng.3913 28714975

[B75] Ni Y. Li J. Panagiotou G. (2015). A molecular-level landscape of diet-gut microbiome interactions: toward dietary interventions targeting bacterial genes. mBio 6, e01263–15. 10.1128/mBio.01263-15 26507230 PMC4626853

[B76] Nikpay M. Goel A. Won H.-H. Hall L. M. Willenborg C. Kanoni S.. (2015). A comprehensive 1,000 genomes-based genome-wide association meta-analysis of coronary artery disease. Nat. Genet. 47, 1121–1130. 10.1038/ng.3396 26343387 PMC4589895

[B77] Pallazola V. A. Davis D. M. Whelton S. P. Cardoso R. Latina J. M. Michos E. D.. (2019). A clinician's guide to healthy eating for cardiovascular disease prevention. Mayo Clin. Proc. Innov. Qual. Outcomes 3, 251–267. 10.1016/j.mayocpiqo.2019.05.001 31485563 PMC6713921

[B78] Pasolli E. Truong D. T. Malik F. Waldron L. Segata N. (2016). Machine learning meta-analysis of large metagenomic datasets: tools and biological insights. PLoS Comput. Biol. 12, e1004977. 10.1371/journal.pcbi.1004977 27400279 PMC4939962

[B79] Qi L.. (2012). Gene-diet interactions in complex disease: current findings and relevance for public health. Curr. Nutr. Rep. 1, 222–227. 10.1007/s13668-012-0029-8 23139897 PMC3489189

[B80] Reel P. S. Reel S. Pearson E. Trucco E. Jefferson E. (2021). Using machine learning approaches for multi-omics data analysis: a review. Biotechnol. Adv. 49, 107739. 10.1016/j.biotechadv.2021.107739 33794304

[B81] Reynolds K. Go A. S. Leong T. K. Boudreau D. M. Cassidy-Bushrow A. E. Fortmann S. P.. (2017). Trends in incidence of hospitalized acute myocardial infarction in the cardiovascular research network (CVRN). Am. J. Med. 130, 317–327. 10.1016/j.amjmed.2016.09.014 27751900 PMC5318252

[B82] Rigdon J. Basu S. (2019). Machine learning with sparse nutrition data to improve cardiovascular mortality risk prediction in the USA using nationally randomly sampled data. BMJ Open 9, e032703. 10.1136/bmjopen-2019-032703 31784446 PMC6924849

[B83] Roger V. L. Sidney S. Fairchild A. L. Howard V. J. Labarthe D. R. Shay C. M.. (2020). Recommendations for cardiovascular health and disease surveillance for 2030 and beyond: a policy statement from the american heart association. Circulation 141, e104–e119. 10.1161/CIR.0000000000000756 31992050

[B84] Samani N. J. Erdmann J. Hall A. S. Hengstenberg C. Mangino M. Mayer B.. (2007). Genomewide association analysis of coronary artery disease. N. Engl. J. Med. 357, 443–453. 10.1056/NEJMoa072366 17634449 PMC2719290

[B85] Sanchez-Rodriguez E. Egea-Zorrilla A. Plaza-Diaz J. Aragón-Vela J. Munoz-Quezada S. Tercedor-Sánchez L.. (2020). The gut microbiota and its implication in the development of atherosclerosis and related cardiovascular diseases. Nutrients 12, 605. 10.3390/nu12030605 32110880 PMC7146472

[B86] Schunkert H. Konig I. R. Kathiresan S. Reilly M. P. Assimes T. L. Holm H.. (2011). Large-scale association analysis identifies 13 new susceptibility loci for coronary artery disease. Nat. Genet. 43, 333–338. 10.1038/ng.784 21378990 PMC3119261

[B87] Schunkert H. von Scheidt M. Kessler T. Stiller B. Zeng L. Vilne B. (2018). Genetics of coronary artery disease in the light of genome-wide association studies. Clin. Res. Cardiol. 107, 2–9. 10.1007/s00392-018-1324-1 30022276

[B88] Solares J. R. A. Raimondi F. E. D. Zhu Y. Rahimian F. Canoy D. Tran J.. (2020). Deep learning for electronic health records: a comparative review of multiple deep neural architectures. J. Biomed. Inform. 101, 103337. 10.1016/j.jbi.2019.103337 31916973

[B89] Spanakis G. Weiss G. Boh B. Lemmens L. Roefs A. (2017). Machine learning techniques in eating behavior e-coaching. Pers. Ubiquit. Comput. 21, 645–659. 10.1007/s00779-017-1022-4

[B90] Spector T. Berry S. Valdes A. Drew D. Chan A. Franks P.. (2019). Integrating metagenomic information into personalized nutrition tools: the PREDICT I study (p20-005-19). Curr. Dev. Nutr. 3(Suppl 1), nzz040.P20-005-19. 10.1093/cdn/nzz040.P20-005-19

[B91] Stephens Z. D. Lee S. Y. Faghri F. Campbell R. H. Zhai C. Efron M. J.. (2015). Big data: astronomical or genomical? PLoS Biol. 13, e1002195. 10.1371/journal.pbio.1002195 26151137 PMC4494865

[B92] Suykens J. A. Vandewalle J. De Moor B. (2001). Optimal control by least squares support vector machines. Neural Netw. 14, 23–35. 10.1016/S0893-6080(00)00077-0 11213211

[B93] Tang W. H. W. Kitai T. Hazen S. L. (2017). Gut microbiota in cardiovascular health and disease. Circ. Res. 120, 1183–1196. 10.1161/CIRCRESAHA.117.309715 28360349 PMC5390330

[B94] Tregouet D.-A. Konig I. R. Erdmann J. Munteanu A. Braund P. S. Hall A. S.. (2009). Genome-wide haplotype association study identifies the slc22a3-lpal2-lpa gene cluster as a risk locus for coronary artery disease. Nat. Genet. 41, 283–285. 10.1038/ng.314 19198611

[B95] Trøseid M. Andersen G. Ø. Broch K. Hov J. R. (2020). The gut microbiome in coronary artery disease and heart failure: current knowledge and future directions. eBiomedicine 52, 102649. 10.1016/j.ebiom.2020.102649 32062353 PMC7016372

[B96] Turnbaugh P. J. Hamady M. Yatsunenko T. Cantarel B. L. Duncan A. Ley R. E.. (2009). A core gut microbiome in obese and lean twins. Nature 457, 480–484. 10.1038/nature07540 19043404 PMC2677729

[B97] van der Harst P. Verweij N. (2018). Identification of 64 novel genetic loci provides an expanded view on the genetic architecture of coronary artery disease. Circ. Res. 122, 433–443. 10.1161/CIRCRESAHA.117.312086 29212778 PMC5805277

[B98] Venkataraman A. Sieber J. R. Schmidt A. W. Waldron C. Theis K. R. Schmidt T. M. (2016). Variable responses of human microbiomes to dietary supplementation with resistant starch. Microbiome 4, 33. 10.1186/s40168-016-0178-x 27357127 PMC4928258

[B99] Verma M. Hontecillas R. Tubau-Juni N. Abedi V. Bassaganya-Riera J. (2018). Challenges in personalized nutrition and health. Front. Nutr. 5, 117. 10.3389/fnut.2018.00117 30555829 PMC6281760

[B100] Vilne B. Meistere I. Grantina-Ievina L. Kibilds J. (2019). Machine learning approaches for epidemiological investigations of food-borne disease outbreaks. Front. Microbiol. 10, 1722. 10.3389/fmicb.2019.01722 31447800 PMC6691741

[B101] Vilne B. Schunkert H. (2018). Integrating genes affecting coronary artery disease in functional networks by multi-omics approach. Front. Cardiovasc. Med. 5, 89. 10.3389/fcvm.2018.00089 30065929 PMC6056735

[B102] Vilne B. Skogsberg J. Foroughi Asl H. Talukdar H. A. Kessler T. Bjorkegren J. L. M.. (2017). Network analysis reveals a causal role of mitochondrial gene activity in atherosclerotic lesion formation. Atherosclerosis 267, 39–48. 10.1016/j.atherosclerosis.2017.10.019 29100060

[B103] Webb T. R. Erdmann J. Stirrups K. E. Stitziel N. O. Masca N. G. D. Jansen H.. (2017). Systematic evaluation of pleiotropy identifies 6 further loci associated with coronary artery disease. J. Am. Coll. Cardiol. 69, 823–836. 10.1016/j.jacc.2016.11.056 28209224 PMC5314135

[B104] Weber I. Achananuparp P. (2016). Insights from machine-learned diet success prediction. Pac. Symp. Biocomput. 21, 540–551. 10.1142/9789814749411_0049 26776216

[B105] Weber I. Achananuparp P. (2016). Insights from machine-learned diet success prediction. Pac. Symp. Biocomput. 21, 540–551. 26776216

[B106] Westerlund A. M. Hawe J. S. Heinig M. Schunkert H. (2021). Risk prediction of cardiovascular events by exploration of molecular data with explainable artificial intelligence. Int. J. Mol. Sci. 22:10291. 10.3390/ijms221910291 34638627 PMC8508897

[B107] Wilson P. W. D'Agostino R. B. Levy D. Belanger A. M. Silbershatz H. Kannel W. B. (1998). Prediction of coronary heart disease using risk factor categories. Circulation 97, 1837–1847. 10.1161/01.CIR.97.18.1837 9603539

[B108] Zeevi D. Korem T. Zmora N. Israeli D. Rothschild D. Weinberger A.. (2015). Personalized nutrition by prediction of glycemic responses. Cell 163, 1079–1094. 10.1016/j.cell.2015.11.001 26590418

[B109] Zhao Z. Yang Y. Zeng Y. He M. (2016). A microfluidic exosearch chip for multiplexed exosome detection towards blood-based ovarian cancer diagnosis. Lab Chip 16, 489–496. 10.1039/C5LC01117E 26645590 PMC4729647

[B110] Zhu Q. Gao R. Zhang Y. Pan D. Zhu Y. Zhang X.. (2018). Dysbiosis signatures of gut microbiota in coronary artery disease. Physiol. Genomics 50, 893–903. 10.1152/physiolgenomics.00070.2018 30192713

